# Preliminary Results on the Prevalence of *Salmonella* spp. in Marine Animals Stranded in Sicilian Coasts: Antibiotic Susceptibility Profile and ARGs Detection in the Isolated Strains

**DOI:** 10.3390/pathogens10080930

**Published:** 2021-07-23

**Authors:** Delia Gambino, Sonia Sciortino, Sergio Migliore, Lucia Galuppo, Roberto Puleio, Salvatore Dara, Domenico Vicari, Salvatore Seminara, Valeria Gargano

**Affiliations:** Istituto Zooprofilattico Sperimentale della Sicilia “A. Mirri”, 90129 Palermo, Italy; deliagamb@gmail.com (D.G.); galuppolucia@gmail.com (L.G.); roberto.puleio@izssicilia.it (R.P.); salvatore.dara@izssicilia.it (S.D.); domenico.vicari@izssicilia.it (D.V.); salvatore.seminara@izssicilia.it (S.S.); valeria.gargano@izssicilia.it (V.G.)

**Keywords:** *Salmonella enterica*, antibiotic resistance, marine animals

## Abstract

The presence of *Salmonella* spp. in marine animals is a consequence of contamination from terrestrial sources (human activities and animals). Bacteria present in marine environments, including *Salmonella* spp., can be antibiotic resistant or harbor resistance genes. In this study, *Salmonella* spp. detection was performed on 176 marine animals stranded in the Sicilian coasts (south Italy). Antibiotic susceptibility, by disk diffusion method and MIC determination, and antibiotic resistance genes, by molecular methods (PCR) of the *Salmonella* spp. strains, were evaluated. We isolated *Salmonella* spp. in three animals, though no pathological signs were detected. Our results showed a low prevalence of *Salmonella* spp. (1.7%) and a low incidence of phenotypic resistance in three *Salmonella* spp. strains isolated. Indeed, of the three strains, only *Salmonella* subsp. enterica serovar Typhimurium from *S. coeruleoalba* and *M. mobular* showed phenotypic resistance: the first to ampicillin, tetracycline, and sulphamethoxazole, while the latter only to sulphamethoxazole. However, all strains harbored resistance genes (*bla*_TEM_, *bla*_OXA_, *tet*(A), *tet*(D), *tet*(E), *sul*I, and *sul*II). Although the low prevalence of Salmonella spp. found in this study does not represent a relevant health issue, our data contribute to the collection of information on the spread of ARGs, elements involved in antibiotic resistance, now considered a zoonosis in a One Health approach.

## 1. Introduction

*Salmonella* are zoonotic pathogens of humans and animals, and the leading cause of foodborne outbreaks and infections in many countries [1]. Studies have focused on *S. enterica*, and particularly on the serovars Typhimurium and Enteritidis, which are the most prevalent in humans and animals and the most linked to infections [1]. Animals can be asymptomatic carriers of *Salmonella* or, similar to humans, can exhibit symptoms ranging from localized intestinal inflammation and gastroenteritis to life-threatening systemic infection. Transmission can occur by direct contact with infected individuals, animals or humans, or most frequently by fecal–oral route. Indeed, the most common reservoir of this microorganism is the intestinal tract of a wide range of domestic and wild animals that can serve as vehicles for *Salmonella* spp. and spread infected feces that can contaminate water and food [2].

Even if the microorganisms of the genus *Salmonella* are not natural inhabitants of aquatic environments, several serovars are widely distributed in water (sea, estuarine, river) and in a variety of seafood [3]. In many cases, aquatic environments, especially coastal environments, receive water from hospitals, farms, and aquaculture wastewater treatment plants, which may contain pathogens from human activities, or directly from rivers, which may carry enteric bacteria from their natural reservoirs inland. As a result, the presence of *Salmonella* spp. in these environments represents a source of contamination for marine animals. Several studies report the presence of *S. enterica* in many species of marine wildlife, such as sea lions (*Zalophus californianusliberi*), elephant seals (*Mirounga angustirostris*), gray seals (*Halichoreus grypus*), sea turtles (*Dermochelys coriacea*) and dolphins (*Stenella coeruleoalba*) [4,5,6,7].

Bacteria, including pathogenic species, found in marine environments may be resistant to antibiotics, or harbor transmissible antibiotic resistance genes. Indeed, several studies conducted on aquatic environments and marine wildlife have shown that these can be reservoirs of antimicrobial resistance (AMR) [8,9,10]. AMR is the bacteria’s ability to resist the effects of an antibiotic to which they were previously sensitive. Bacteria acquire this ability through genetic mutations, or from the acquisition of antibiotic resistance genes (ARGs). When this phenomena occurs in pathogenic strains, causing infection, there are higher mortality rates, and it is estimated that infections with resistant bacteria will cause 10 million deaths worldwide by the year 2050 [11]. For these reasons, the World Health Organization (WHO) published a list of priority antibiotic-resistant pathogens, which includes *Salmonella* spp. and other families of bacteria considered to pose the greatest threat to human health [12]. In addition, the latest data on humans, animals, and food show that a large percentage of *Salmonella* strains are multi-drug resistant (resistant to three or more antimicrobials) and harbor ARGs [13].

In Italy, the Ministry of the Environment and the Ministry of Health have introduced a system to monitor the beaching of cetaceans and sea turtles on the Italian coast. The Istituto Zooprofilattico Sperimentale della Sicilia is one of the competent units which, after receiving notifications of stranded animals from the Port Authority, proceeds to recuperate the carcasses and carry out examinations (necropsies and laboratory analyses) to collect data on their state of health and establish the causa mortis. However, the state of preservation of carcasses does not always allow the cause of death to be established, and often the advanced putrefaction of carcasses makes pathogen isolation by culture methods difficult.

In the present study, we report the presence of *Salmonella* spp. in a specimen of loggerhead sea turtle (*Caretta caretta*), striped dolphin (*Stenella coeruleoalba*), and manta ray (*Mobula mobular*), respectively, found dead in Sicilian coastal waters. Due to the pathogenic potential and possible AMR of this microorganism [14], the antibiotic susceptibility profile and, subsequently, the presence of genetic elements of resistance were also evaluated. In fact, even if the prevalence of *Salmonella* spp. is not sufficiently documented to represent a health problem, the detection of ARGs in strains isolated from these animals could contribute to the collection of data on the dissemination of AMR, which is now considered a zoonosis in a One Health approach.

## 2. Materials and Methods

### 2.1. Sample Collection

**Figure 1:**
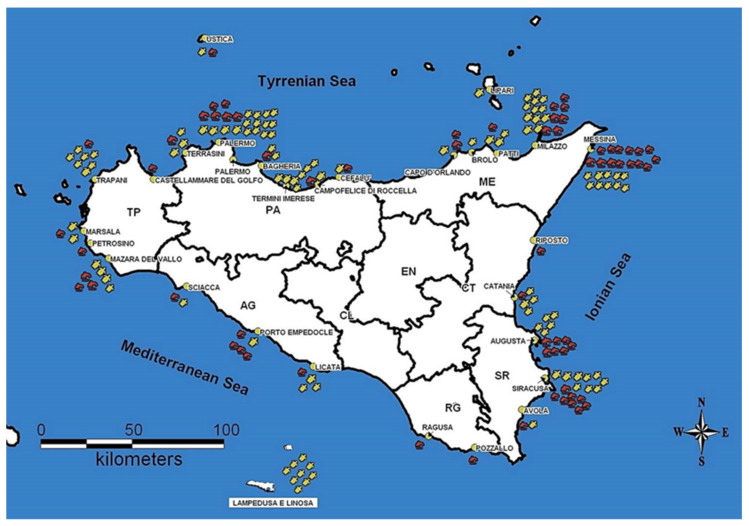
Finding locations of the analyzed subjects. TP: Trapani; PA: Palermo; ME: Messina; AG: Agrigento; CL: Caltanissetta; EN: Enna; CT: Catania; RG: Ragusa; SR: Siracusa. Red symbols represent cetaceans and yellow symbols represent sea turtles.

In the years from 2016 to 2020, the carcasses of 107 sea turtles *C. caretta*, 68 cetaceans (56 *S. coeruleoalba*, 11 *Tursiops truncatus*, and 1 *Grampus griseus*) and 1 *M. mobular* were analyzed. All recovered subjects were found dead in the Sicilian coasts, and mainly in the provinces of Palermo and Messina. In order to investigate aspects of the health status of the recovered specimens, within 48 h of their arrival all carcasses were subjected to necropsy and, according to the state of preservation, tissue samples for laboratory analysis were collected. The finding location of the analyzed subjects are shown in Figure 1.

### 2.2. Isolation of Salmonella spp.

Detection of *Salmonella* spp. was conducted on cloacal/anal swabs and on gastrointestinal tract (spleen, liver, and intestine) from 176 animals. For the isolation, the samples were pre-enriched in buffered peptone water, and subsequently enriched in Selenite Cystine and Rappaport Vassiliadis broth, then incubated, respectively, at 37 °C and 42 °C. After incubation for 24 h, both broths were seeded on Xylose Lysine Desoxycholate and Brilliant Green agar [2]. Colonies that showed growth characteristics attributable to the genus *Salmonella* were transplanted into nutrient agar and subjected to biochemical-enzymatic tests for genus identification. All the media were purchased from Oxoid (Milano, Italy).

Strains identified as *Salmonella* spp. were sent to the National Reference Center and OIE Reference Laboratory for Salmonellosis of the Istituto Zooprofilattico Sperimentale delle Venezie (IZSVe, Padova, Italy) for serotyping.

### 2.3. Antimicrobial Susceptibility Using the Disk Diffusion Method

The antibiotic susceptibility profile was determined using the disk diffusion method according to Clinical and Laboratory Standard Institute (CLSI) standards [15]. A panel that included antibiotics representative of the six most used antibiotic classes was chosen. The antibiotics tested were: ampicillin (AMP, 10 µg), gentamicin (GEN, 10 µg), cefotaxime (CTX, 30 µg), chloramphenicol (CHL, 10 µg), nalidixic acid (NAL, 30 µg), ciprofloxacin (CIP, 5 µg), sulfamethoxazole + trimethoprim (SXT, 25 µg), and tetracycline (TET, 30 µg).

The strains were classified as resistant (R), intermediate (I) or susceptible (S) in accordance with CLSI ranges [15]. Antimicrobial disks were purchased from Oxoid (Milano, Italy).

### 2.4. Determination of Minimum Inhibitory Concentration (MIC)

Minimum Inhibitory Concentration (MIC) values (µg/mL) were determined for the 3 *Salmonella* strains using a plate-based microdilution method. The antimicrobial susceptibility commercial plate (96-well Sensititre ™ EU Surveillance *Salmonella/E. coli* EUVSEC Plate Thermo Scientific, Waltham, MA, USA) contained scaled dilutions (2-fold dilutions) for the following 14 antimicrobials: sulfamethoxazole (SMX, 8–1024), trimethoprim (TMP, 0.25–32), ciprofloxacin (CIP, 0.015–8), tetracycline (TET, 2–64), meropenem (MERO, 0.03–16), azithromycin (AZI, 2–64), nalidixic acid (NAL, 4–128), cefotaxime (FOT, 0.25–4), chloramphenicol (CHL, 8–128), tigecycline (TGC, 0.25–8), ceftazidime (TAZ, 0.5–8), colistin (COL, 1–16), ampicillin (AMP, 1–64), and gentamicin (GEN, 0.5–32).

In accordance with the manufacturer’s instructions, the inoculum was prepared from a bacterial suspension in sterile water of 0.5 McF and Mueller–Hinton broth. Plates were inoculated and incubated in an aerobic atmosphere at 37 °C for 18–24 h. After the incubation time, a manual reading was performed using Sensititre ™ Manual Viewbox (Thermo Scientific, USA) and the results were interpreted in accordance with CLSI breakpoints [15].

### 2.5. Detection of Antibiotic Resistance Genes and Class-1 Integron

The presence of resistance genes to some of the most widely used antibiotics for salmonellosis therapy (beta-lactams, tetracyclines, and sulphonamides) and the mobile element *int1* was determined by single PCR following previously published protocols [16]. Subsequently, the amplicons size was determined by electrophoresis on a 2% agarose gel. The targets of ARGs investigated in this study were reported in Table 1. A short portion of 16S rDNA gene was used as positive control and nuclease-free water for molecular biology as negative control. Furthermore, one amplicon from each gene was sequenced to confirm the specificity of the PCR reaction by previously published protocols [17].

## 3. Results

### 3.1. Isolation and Identification of Salmonella spp.

Bacteriological analysis performed on gastrointestinal tract organs (spleen, liver, and intestine) and cloacal/rectal swab of 176 marine animals allowed to the isolation of three strains of *Salmonella* spp. A prevalence of *Salmonella* spp. of 1.7% was found; in particular, the three strains were found in 2016, 2018, and 2020 with a prevalence per year of 1.7% (1/59), 3% (1/33), and 3.7% (1/27), respectively. The serotyping test showed that the three strains belonged to *enterica* species and, in particular, the two strains isolated from *S. coeruleoalba* and *M. mobular* to the Typhimurium 4, 12; i; 1,2 serovars (*S.* Typhimurium), while the strain from *C. caretta* to the Enteritidis 1, 9, 12; g, m; - serovar (*S.* Enteritidis). 

These three strains were isolated from the spleen of a *S. coeruleoalba* (found in Augusta, Siracusa) and a *C. caretta *(found in Trapani), and from the rectal swab of *M. mobular* (found in Palermo). These animals had no lesions attributable to *Salmonella* spp. and no comorbidities. Notably, the sea turtle, a female with a 50 cm long and 43 cm wide carapace, was found with a fishing line around the intestine and hook related cuts on the internal organs. The striped dolphin, a female 1.65 m long and 45 kg in weight, had a perforation at the base of the dorsal fin, probably caused by a harpoon, and signs of asphyxiation in the lungs. Finally, the manta ray, a female 3.78 m long and 3.3 m wide, showed an injury on the dorsal side possibly related to a collision with a boat.

### 3.2. Antibiotic Susceptibility Results

The disk diffusion method showed a low incidence of resistance in these three strains. Indeed, only the *S.* Typhimurium strain isolated from the striped dolphin showed resistance to ampicillin and tetracycline. This result was confirmed by the quantitative method (MIC); indeed, this strain showed for both antibiotics MIC ≥ 64 µg. In addition, the MIC revealed that both strains of *S.* Typhimurium, from the striped dolphin and the manta ray were resistant to sulphamethoxazole (MIC ≥ 1024), but susceptible to trimethoprim (MIC ≤ 0.25).

### 3.3. Detection of Antibiotic Resistance Genes and Int1

Molecular analysis conducted for the detection of β-lactamases (*bla*_TEM_, *bla*_CTXM_, *bla*_SHV_, and *bla*_OXA_), tetracyclines (*tet*(A), *tet*(B), *tet*(C), *tet*(D), *tet*(E), *tet*(G), *tet*(O), and *tet*(W)), and sulphonamides (*sul*I, *sul*II, and *sul*III) resistance genes showed that all three *Salmonella* spp. strains harbored multiple genes among those screened for Table 2. Concerning β-lactamases genes, only *bla*_TEM_*,* hosted by two strains (*S.* Enteritidis from the sea turtle and *S.* Typhimurium from the manta ray), and *bla*_OXA_, hosted by *S.* Typhimurium from the striped dolphin, were detected. In contrast, tetracyclines and sulphonamides resistance genes were detected in all strains. Indeed, *S.* Typhimurium from the dolphin harbored *tet*(A), *tet*(E), and *sul*II; *S.* Enteritidis from the sea turtle harbored *tet*(D), *tet*(E), and *sul*I; and *S.* Typhimurium from the manta ray harbored *tet*(A), *tet*(D), *tet*(E), *sul*I, and *sul*II. In addition, the presence of the mobile element *int1* was also detected in the *Salmonella* strain isolated from *M. mobular.*

## 4. Discussion

In this study, we report the isolation of *Salmonella* spp. strains in three marine animals belonging to three different classes: Reptilia, Mammalia, and Fish, confirming the broad-host-range of this bacterium and its diffusion in Mediterranean Sea.

The 176 individuals analyzed belong to species on the International Union for Conservation of Nature (IUCN) Red Lists with different conservation statuses. *S. coeruleoalba* and *G. griseus* are among the species for which there is less concern, while *C. caretta* and *T. truncatus* are considered vulnerable, and *M. mobular* is considered an endangered species. Dolphin and sea turtle species analyzed are widespread in the Mediterranean Sea, and they are predominantly pelagic species, although occasionally are sighted near the coast, whereas *M. mobular* is an Atlanto–Mediterranean species that spends most of its life away from the mainland [23,24,25,26,27]. While specimens of *S. coeruleoalba* and *C. caretta*, especially in recent years, have been frequently rescued in the Italian coasts, the same is not true for *M. mobular*, whose sightings in Italy are rare.

Other studies report the presence of *Salmonella* spp. in marine animals, even in sites far from human activities, with increasing frequency in the last decade [5,7,28]. However, even if many marine species are known to harbor *S. enterica*, reports of *Salmonella*-associated illness in wild marine animals are rare, and usually occur in debilitated or stressed individuals [29]. In addition, the increased frequency of isolation from marine environments may be related to the increased number of studies conducted on bacterial populations in marine animals, but may also be the result of the increasing human activity or of the exposure between marine wildlife and other infected animals, marine or terrestrial [30]. The strains isolated in this study belong to the Typhimurium and Enteritidis serovars, that are among the most commonly reported serovars in European Union, and are isolated both in cases of salmonellosis in humans and from farm animals, such as chickens, pigs, turkeys, and cattle [1].

Antibiotic susceptibility tests showed low incidence of phenotypic resistances in the *Salmonella* spp. strains analyzed. Indeed, the *S.* Typhimurium strain from *S. coeruleoalba* showed resistance to three antibiotics, ampicillin, tetracycline, and sulfamethoxazole, while the *S.* Typhimurium strain from the manta ray was resistant only to sulfamethoxazole. Resistance to sulfonamides, tetracyclines, and ampicillin is frequently reported in *Salmonella* isolates from humans, animals (food-producing and pets), and food of animal origin [13]. Moreover, *Salmonella* resistant to these antibiotics have also been found in shellfish, indicating the spread of antibiotic-resistant strains from human or animal feces to aquatic ecosystems [31].

However, despite the low incidence of phenotypic resistance, molecular analysis highlighted that these three strains all harbored at least one resistance gene for each of the four widely used classes of antibiotics: β-lactamases (*bla*_TEM_ and *bla*_OXA_), tetracyclines (*tet*(A), *tet*(D), and *te*t(E)), and sulfonamides (*sul*I and *sul*II). The presence of these genes is reported in both phenotypically susceptible and resistant strains, but in order to contribute to antibiotic resistance the genes must be expressed [17,32,33]. Furthermore, ARGs are normally found linked to other resistance genes in integrons or on plasmids, and can therefore be transferred to other strains. In natural environments, including the marine environment, the high abundance and density of bacteria and the presence of optimal conditions facilitate the transfer of genetic determinants, while high selection pressure, resulting from the presence of sub-inhibitory concentrations of antibiotics, metals, and toxic materials, can induce gene expression and therefore phenotypic resistance [34,35].

## 5. Conclusions

Our data showed a low prevalence of Salmonella spp. in marine animals stranded on the Sicilian coast, nevertheless highlighting the presence of ARGs in the isolated strains. Further health and epidemiological studies to determine the origin and role of Salmonella spp. in these animals are needed. In conclusion, our data contribute to increase the information on the spread of ARGs in the marine environment, highlighting the importance of marine animals as environment indicators, useful for the protection and conservation of marine habitats, and not least for the public health preservation according to the “One Health” approach. 

## Figures and Tables

**Table 1:** Primers used in this study.

Target Name	Resistance Mechanism	Amplicon Size (bp)	Reference
bla_TEM_	β-Lactamases	661	[18]
bla_CTXM_	β-Lactamases	585	[18]
bla_PSE_	β-Lactamases	575	[18]
bla_SHV_	β-Lactamases	807	[18]
bla_OXA_	β-Lactamases	590	[18]
*tet*(A)	Efflux	210	[16]
*tet*(B)	Efflux	659	[19]
*tet*(C)	Efflux	418	[19]
*tet*(D)	Efflux	787	[20]
*tet*(E)	Efflux	278	[20]
*tet*(G)	Efflux	468	[20]
*tet*(O)	Ribosomal protection	88	[21]
*tet*(W)	Ribosomal protection	120	[21]
*sul*I	Dihydropteroate synthase inhibitor	316	[19]
*sul*II	Dihydropteroate synthase inhibitor	191	[16]
*sul*III	Dihydropteroate synthase inhibitor	799	[19]
*int1*	Class 1 integron	148	[16]
16S rDNA	Positive control	142	[22]

**Table 2:** Results of ARGs detection.

Isolated Strains	Animals	Phenotypic Resistance	β-Lactamases	Tetracyclines	Sulphonamides	*Int1*
*S.* Typhimurium	*S. coeruleoalba*	AMP, TET, SMX	*bla*_OXA_	*tet*(A)*, tet*(E)	*sul*II	
*S.* Enteritidis	*C. caretta*	*	*bla*_TEM_	*tet*(D)*, tet*(E)	*sul*I	
*S.* Typhimurium	*M. mobular*	SMX	*bla*_TEM_	*tet*(A)*, tet*(D)*, tet*(E)	*sul*I, *sul*II	*int1*

AMP: ampicillin; TET: tetracycline; and SMX: sulfamethoxazole; * no resistance to antibiotics tested.

## Data Availability

All data discussed are contained in the article.
